# Success of trial of labor in women with a history of previous cesarean section for failed labor induction or labor dystocia: a retrospective cohort study

**DOI:** 10.1186/s12884-019-2334-3

**Published:** 2019-05-20

**Authors:** Katariina Place, Heidi Kruit, Aydin Tekay, Seppo Heinonen, Leena Rahkonen

**Affiliations:** Department of Obstetrics and Gynecology, University of Helsinki and Helsinki University Hospital, Haartmaninkatu 2, 00029 HUS, Helsinki, Finland

**Keywords:** Cesarean section, Induction of labor, Labor dystocia, Trial of labor after cesarean (TOLAC)

## Abstract

**Background:**

The rates of cesarean section (CS) are increasing worldwide leading to an increased risk for maternal and neonatal complications in the subsequent pregnancy and labor. Previous studies have demonstrated that successful trial of labor after cesarean (TOLAC) is associated with the least maternal morbidity, but the risks of unsuccessful TOLAC exceed the risks of scheduled repeat CS. However, prediction of successful TOLAC is difficult, and only limited data on TOLAC in women with previous failed labor induction or labor dystocia exists. Our aim was to evaluate the success of TOLAC in women with a history of failed labor induction or labor dystocia, to compare the delivery outcomes according to stage of labor at time of previous CS, and to assess the risk factors for recurrent failed labor induction or labor dystocia.

**Methods:**

This retrospective cohort study of 660 women with a prior CS for failed labor induction or labor dystocia undergoing TOLAC was carried out in Helsinki University Hospital, Finland, between 2013 and 2015. Data on the study population was obtained from the hospital database and analyzed using SPSS.

**Results:**

The rate of vaginal delivery was 72.9% and the rate of repeat CS for failed induction or labor dystocia was 17.7%. The rate of successful TOLAC was 75.6% in women with a history of labor arrest in the first stage of labor, 73.1% in women with a history of labor arrest in the second stage of labor, and 59.0% in women with previous failed induction. The adjusted risk factors for recurrent failed induction or labor dystocia were maternal height < 160 cm (OR 1.9 95% CI 1.1–3.1), no prior vaginal delivery (OR 8.3 95% CI 3.5–19.8), type 1 or gestational diabetes (OR 1.8 95% CI 1.0–3.0), IOL for suspected non-diabetic fetal macrosomia (OR 10.8 95% CI 2.1–55.9) and birthweight ≥4500 g (OR 3.3 95% CI 1.3–7.9).

**Conclusions:**

TOLAC is a feasible option to scheduled repeat CS in women with a history of failed induction or labor dystocia. However, women with no previous vaginal delivery, maternal height < 160 cm, diabetes or suspected neonatal macrosomia (≥4500 g) may be at increased risk for failed TOLAC.

## Background

The rates of cesarean section (CS) are increasing worldwide. In Finland, the rate of CS is 16% of all births, and 20% in primiparous women, being the lowest figures among the western countries [1]. Since CS leads to an increased risk for abnormally attached placenta, uterine rupture, and maternal and neonatal complications in the subsequent pregnancy and labor [2], an abundance of research has been conducted to assess the feasibility and safety of trial of labor after cesarean (TOLAC) [3, 4].

Previous studies suggest that the mode of delivery with the least maternal morbidity for a woman with a history of prior low transverse CS is successful TOLAC [5–7], but the risks of unsuccessful TOLAC are higher than the risks of scheduled repeat CS [6]. However, prediction of successful TOLAC is difficult. TOLAC is suggested to be cost-effective compared to a repeat planned CS [8], and when considering long-term consequences, TOLAC with a success rate of 47% or more appears more economical than a repeat planned CS [9].

As shown by previous studies, the indication of the previous CS influences the success rate of TOLAC. Women with a previous CS for labor dystocia (non-progressing labor) have a lower rate of successful TOLAC compared to women with a nonrecurring CS indication such as breech presentation or fetal distress [10, 11]. The greatest predictor for successful TOLAC is a prior vaginal delivery [4, 12]. Prelabor nomograms have been presented to predict successful TOLAC [13, 14], but the data on induction of labor (IOL) in women with a history of previous CS for failed induction or labor dystocia is limited [15].

Our primary aim was to evaluate the success of TOLAC, including IOL, in this subgroup of women, and to compare delivery outcomes according to stage of labor at time of the previous CS. We also wanted to assess risk factors for repeat CS for recurrent failed labor induction or labor dystocia.

## Methods

**Fig. 1:**
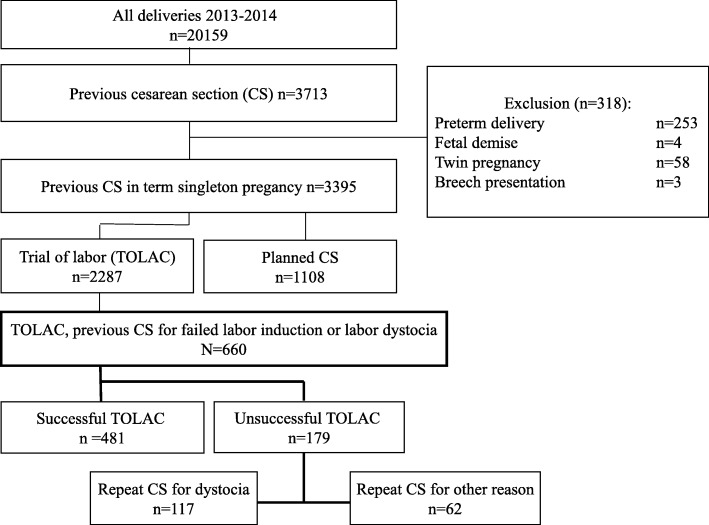
Flow chart of the study population

This retrospective cohort study of women with a history of previous emergency CS for labor dystocia or failed labor induction was carried out in the Department of Obstetrics and Gynecology of Helsinki University Hospital, Finland. All women with a vital singleton term pregnancy, cephalic presentation, and previous lower segment transverse CS for failed labor induction or labor dystocia undergoing TOLAC between January 1st 2013 and January 1st 2015 were identified in the hospital database. Women with scheduled repeat CS, preterm delivery, breech presentation, twin pregnancy or fetal demise were excluded (Fig. 1). Women undergoing IOL and women with spontaneous onset of labor were both included in the study (Fig. 1). The study protocol was approved by the management of Hospital district of Helsinki and Uusimaa. An informed consent was not required since this was a retrospective cohort study approved by the hospital management.

Data on the study population characteristics and labor and delivery outcomes were obtained from individual patient charts in the hospital database.

Gestational age was determined by the crown-rump-length measurement at the time of the first trimester ultrasound screening. Obesity was defined as BMI ≥35 kg/m^2^, and gestational diabetes was diagnosed by a 2-h 75 g oral glucose tolerance test. Medication-dependent gestational diabetes included both insulin and metformin treatments. Post-term pregnancy was defined as gestational age ≥ 42^+ 0^ weeks. Fetal macrosomia was defined by birthweight ≥4500 g. In case of term premature rupture of membranes (PROM), labor was induced after 24 h of expectant management.

The indications for labor induction were categorized as post-term pregnancy, PROM, gestational diabetes, suspected non-diabetic fetal macrosomia, fetal reason and maternal reason. Fetal reason for labor induction included oligohydramnios, polyhydramnios, nonreassuring cardiotocograph, intrauterine growth restriction, Rh-immunization, reduced fetal movements, prevention of fetal malposition after successful external cephalic version, and gastrointestinal anomalies. Maternal reason for labor induction included pre-eclampsia, intrahepatic cholestasis of pregnancy, maternal medical condition, exhaustion, psychosocial reasons, and complications in an earlier pregnancy such as previous intrauterine death or shoulder dystocia.

Labor induction was carried out by artificial rupture of membranes (amniotomy) and oxytocin in case of a favorable cervix (Bishop score ≥ 6). In case of an unfavorable cervix, a single 50 ml Foley catheter (FC, Rüsh 2-way Foley, Couvelaire tip, catheter size 22Ch, Teleflex Medical, Athlone, Ireland) or misoprostol (Cytotec, Piramal Healthcare UK Limited, Northumberland, England) were used for cervical ripening. FC was retained for a maximum of 24 h. Misoprostol was administered 50 μg orally or 25 μg vaginally every 4 h until Bishop score ≥ 6 was achieved. Amniotomy was performed when the cervix was favorable, and oxytocin was started after 2–12 h if contractions deemed inadequate. Continuous fetal cardiotocography was routinely used during labor.

Failed labor induction was defined as failure to progress in the setting of ruptured membranes, oxytocin infusion for ≥12 h, and cervical dilation < 6 cm [16]. Labor dystocia in the first stage of labor was defined as failure to progress at cervical dilation ≥6 cm with ruptured membranes and adequate contractions for a minimum of 4 h [16]. Labor dystocia in the second stage of labor was defined as failure to deliver at cervical dilation of 10 cm despite of ≥1 h of active pushing or failed operative vaginal delivery [16].

The primary outcomes were the rate of repeat CS and the rate of recurring failed labor induction or labor dystocia. The secondary outcomes were the rates of uterine rupture, postpartum hemorrhage ≥1000 ml, maternal intrapartum and postpartum infections, and adverse neonatal primary outcome [17, 18].

Adverse neonatal primary outcome was defined as a 5-min Apgar-score < 7, umbilical artery blood pH-value < 7.05, or base excess (BE) value <− 12 [17, 18]. Uterine rupture was defined as a complete rupture of both myometrium and visceral peritoneum. Intrapartum infection was defined as fever ≥38 °C, fetal tachycardia, and total white cell count ≥20 e9/l. Postpartum infection included endometritis (defined as fever ≥38 °C, total white cell count ≥20 e9/l, uterine tenderness, and purulent vaginal discharge), clinical wound infection, and urinary tract infection verified by a positive finding in urine culture.

Statistical analysis was performed by using IBM SPSS Statistics for Windows, Version 25.0 (Armonk, NY, USA). Data with categorical variables were compared by Pearson’s Chi-square test. Unpaired comparisons of continuous variables were carried out by Student’s t-test when the data were normally distributed. Multivariate logistic regression analyses were performed to assess relative risks for unsuccessful TOLAC and recurrent CS for failed labor induction or labor dystocia. Adjusted odd ratios (OR) with 95% confidence intervals (CI) were calculated by modelling the data to control for possible confounding factors. All variables used in the multivariate analyses are shown in the tables with respective univariate analyses. A *p*-value < 0.05 was considered statistically significant.

## Results

A total of 660 women with a prior CS for failed labor induction or labor dystocia were included. Of the women, 226 (34.2%) underwent IOL and 434 (65.8%) women had spontaneous onset of labor. A total of 481 women (72.9%) had successful TOLAC. Table 1 presents the characteristics of the study population. The women with unsuccessful TOLAC were shorter, more obese, more often had post-term pregnancy, diabetes, no prior vaginal delivery, and more often underwent IOL (Table 1). A total of 519 women (78.6%) had no prior vaginal delivery, 26 women (3.9%) had delivered vaginally prior to the CS, 106 women (16.1%) had delivered vaginally after the CS, and nine women (1.4%) had prior vaginal delivery both prior to and after the CS. The most common indication for IOL in the current pregnancy was post-term pregnancy (*n* = 65, 28.8%) (Table 1). FC was used as the primary method for IOL in 135 (59.7%) women, misoprostol in 20 (8.8%) women, and amniotomy and oxytocin in 71 (31.4%) women.

**Table 1:** Characteristics of the study population in trial of labor after cesarean (TOLAC) *n* = 660

	Successful TOLAC		Unsuccessful TOLAC		
	*n* = 481	%	*n* = 179	%	*p*
Maternal age ≥ 37	77	16.0	38	21.2	0.12
IVF	13	2.7	3	1.7	0.45
Smoking	33	6.9	15	8.4	0.5
Height < 160 cm	97	20.2	50	27.9	0.03
BMI ≥ 35	34	7.1	19	10.6	0.01
Diabetes	121^1^	25.2	63^2^	35.2	0.01
Prior vaginal delivery	124	25.8	17	9.5	< 0.001
More than 2 years from previous CS	289	60.1	117	65.4	0.22
Medication-dependent gestational diabetes	25	5.5	14	8.5	0.64
Post-term pregnancy (≥42 weeks)	35	7.3	29	16.2	0.001
Labor induction	143	29.7	83	46.4	< 0.001
Bishop < 6 at the start of induction	95	66.4	58	69.8	0.59
Indication for labor induction					
Post-term	37	25.9	28	33.7	0.21
PROM	27	18.9	17	20.5	0.77
Diabetes	28^3^	19.6	12^4^	14.5	0.33
Non-diabetic macrosomia	3	2.1	4	4.8	0.26
Fetal reason	8	5.6	8	9.6	0.25
Maternal reason	40	28.0	14	16.9	0.06

^1^of which 4 women with diabetes type 1 and 117 with gestational diabetes

^2^of which 1 woman with diabetes type 1 and 62 with gestational diabetes

^3^of which 3 women with diabetes type 1 and 25 with gestational diabetes

^4^of which all women with gestational diabetes

The delivery outcomes are presented in Tables 2 and 3. Four cases (0.6%) of uterine rupture occurred (Tables 2 and 3), all in women with no prior vaginal delivery. Three of the uterine ruptures occurred following spontaneous onset of labor and one case occurred following amniotomy and oxytocin induction. There were no cases of hysterectomy. The overall rate of maternal intrapartum infection was 2.9% and postpartum infection 2.4% (Tables 2 and 3). Intrapartum infections and postpartum hemorrhage more often occurred following unsuccessful TOLAC compared to successful TOLAC (Table 2). The rates of oxytocin use (92.9% vs. 89.9%, *p* = 0.21) and epidural or spinal analgesia (86.3% vs. 89.4%, *p* = 0.29) were similar. No significant difference in the rates of adverse primary neonatal outcomes was seen between the groups (Tables 2 and 3). Seventeen (2.6%) neonates had an umbilical artery blood pH < 7.05 at birth, and 26 (3.9%) had a 5-min Apgar score < 7.

**Table 2:** Maternal and neonatal outcomes in successful and unsuccessful trial of labor after cesarean (TOLAC) (*n* = 660)

	Successful TOLAC		Unsuccessful TOLAC		
	*n* = 481	%	*n* = 179	%	*p*-value
Uterine rupture	0	0.0	4	2.2	
Intrapartum infection	4	0.8	15	8.4	< 0.001
Postpartum infection	10	2.1	6	3.4	0.344
Blood culture positive septicemia	1	0.2	0	0	
Postpartum hemorrhage ≥1000 ml	72	15.0	54	30.2	< 0.001
Birthweight ≥4500 g	16	3.3	13	7.3	0.028
Adverse neonatal primary outcome	33^1^	6.9	16^2^	8.9	0.37

^1^missing values in data: pH *n* = 1, BE *n* = 57

^2^missing values in data: pH *n* = 2, BE *n* = 33

**Table 3:** Delivery outcomes in the subgroups of women with a history of labor dystocia in the first stage of labor, labor dystocia in the second stage of labor and failed labor induction (*n* = 660)

	Labor arrest in the 1st stage		Labor arrest in the 2nd stage		Failed labor induction		
	*n* = 508	%	*n* = 52	%	*n* = 100	%	*p*
IOL in current pregnancy	127	25.0	22	42.3	77	77	< 0.001
Mode of delivery							
Spontaneous vaginal delivery	318	62.6	30	57.7	42	42	0.001
Operative vaginal delivery	66	13.0	8	15.4	17	17	0.54
Cesarean section	124	24.4	14	26.9	41	41	0.003
Cesarean section indication							
Labor arrest in the 1st stage	49	9.6	5	9.6	8	8	0.07
Labor arrest in the 2nd stage	24	4.7	4	7.7	1	1	0.007
Failed labor induction	8	1.6	0	0	18	18	< 0.001
Fetal distress	37	7.3	2	3.8	9	9	0.33
Other	6^1^	1.2	3^2^	5.8	5^3^	5	0.04
Postpartum hemorrhage ≥1000 ml	89	17.5	15	28.8	22	22	0.1
Intrapartum infection	11	2.2	2	3.8	6	6	0.07
Postpartum infection	10	2.0	0	0	6	6	0.04
Blood culture positive septicemia	1	0.2	0	0	0	0	1
Uterine rupture	3	0.6	0	0	1	1.0	0.65
Birthweight ≥4500 g	21	4.1	2	3.8	6	6	0.62
Adverse neonatal primary outcome	39	7.7	1	1.9	9	9	0.27

^1^ suspicion of uterine rupture/dehiscence *n* = 1, intrapartum infection *n* = 2, maternal request for fear of labor *n* = 2, fetal malpresentation *n* = 1

^2^ intrapartum infection *n* = 2, maternal request for fear of labor *n* = 1

^3^ intrapartum infection *n* = 3, maternal request for fear of labor *n* = 1, preeclampsia *n* = 1

The rate of successful TOLAC was 75.6% in women with a history of labor arrest in the first stage of labor, 73.1% in women with a history of labor arrest in the second stage of labor, and 59.0% in women with a history of failed labor induction (Table 3). The overall rate of recurring failed labor induction or labor dystocia leading to repeat CS was 17.7%, being 15.9% in women with a history of labor arrest in the first stage of labor, 17.3% in women with a history of labor arrest in the second stage of labor, and 27.0% in women with a history of failed labor induction (*p* = 0.01) (Table 3). Infections were more common in the subgroup of women with previous failed labor induction (Table 3). Seventy-seven women with a history of failed labor induction underwent IOL also in the current pregnancy (Table 3). Of these, 40 women (51.9%) had a successful TOLAC, 26 women (33.8%) had repeat CS for labor dystocia, and 18 (23.4%) women had recurrent failed IOL.

The risk factors for unsuccessful TOLAC are presented in Table 4, and the risk factors for recurring failed labor induction or labor dystocia are presented in Table 5. After adjustment, maternal height < 160 cm (OR 1.9), no prior vaginal delivery (OR 8.3), type 1 or gestational diabetes (OR 1.8), IOL for suspected non-diabetic fetal macrosomia (OR 10.8) and birthweight ≥4500 g (OR 3.3) remained significant risk factors for recurrent failed induction or labor dystocia and repeat CS (Table 5). In the subgroup of women with a history of previous failed labor induction, the adjusted risk factors for recurrent failed labor induction or labor dystocia were smoking (OR 10.5), BMI ≥35 (OR 5.3), and induction of labor for reasons other than PROM or diabetes (Table 6).

**Table 4:** Risk factors for unsuccessful trial of labor after cesarean (TOLAC) (*n* = 179)

	Unadjusted unsuccessful TOLAC			Adjusted unsuccessful TOLAC		
	OR	CI (95%)	*p*-value	OR	CI (95%)	*p*-value
Maternal age ≥ 37	1.4	0.9–2.2	0.12	1.6	1.0–2.7	0.05
IVF	1.6	0.5–5.8	0.45	0.5	0.1–2.1	0.36
Smoking	1.2	0.7–2.3	0.51	1.4	0.7–2.9	0.35
Height < 160 cm	1.5	1.0–2.3	0.03	1.7	1.1–2.7	0.01
BMI ≥ 35	1.6	0.9–2.8	0.14	1.5	0.8–2.9	0.21
No prior vaginal delivery	3.3	1.9–5.7	< 0.001	4.9	2.7–9.0	< 0.001
More than 2 years from previous CS	1.3	0.9–1.8	0.22	1.6	1.1–2.4	0.02
Diabetes, type 1 or gestational	1.6	1.1–2.3	0.01	1.7	1.0–2.6	0.03
Medication-dependent gestational diabetes	1.5	0.8–3.0	0.21	2.1	0.9–5.2	0.11
Post-term pregnancy (≥42 weeks)	2.5	1.5–4.2	0.001	1.5	0.6–4.0	0.43
Labor induction for post-term pregnancy	2.2	1.3–3.8	0.003	1.5	0.6–4.0	0.43
Labor induction for premature rupture of membranes	1.8	0.9–3.3	0.08	2.1	1.0–4.1	0.04
Labor induction for diabetes	1.2	0.6–2.3	0.67	0.7	0.3–1.8	0.47
Labor induction for non-diabetic macrosomia	3.6	0.8–16.4	0.09	5.9	1.2–29.2	0.03
Labor induction for fetal reason	2.8	1.0–7.5	0.05	4.2	1.5–12.2	0.007
Labor induction for maternal reason	0.94	0.5–1.8	0.84	1.3	0.7–2.6	0.46
Birthweight ≥4500 g	2.3	1.1–4.8	0.03	2.4	1.1–5.5	0.03

**Table 5:** Risk factors for recurrent cesarean section for failed labor induction or labor dystocia (*n* = 117)

	Unadjusted recurrent failed induction or labor dystocia			Adjusted recurrent failed induction or labor dystocia		
	OR	CI (95%)	*p*-value	OR	CI (95%)	*p*-value
Maternal age ≥ 37	1.2	0.7–2.1	0.47	1.4	0.8–2.6	0.23
IVF	1.0	0.3–3.4	0.93	0.88	0.2–3.6	0.85
Smoking	1.3	0.6–2.7	0.53	1.8	0.8–4.1	0.18
Height < 160 cm	1.6	1.0–2.6	0.04	1.9	1.1–3.1	0.01
BMI ≥ 35	1.6	0.8–3.2	0.15	1.6	0.8–3.4	0.22
No prior vaginal delivery	5.5	2.5–12.0	< 0.001	8.3	3.5–19.8	< 0.001
More than 2 years from previous CS	1.0	0.7–1.5	0.96	1.3	0.8–2.0	0.31
Diabetes, type 1 or gestational	1.7	1.1–2.6	0.02	1.8	1.0–3.0	0.04
Medication-dependent gestational diabetes	1.5	0.7–3.4	0.3	1.8	0.6–5.3	0.27
Post-term pregnancy (≥42 weeks)	2.3	1.3–4.3	0.007	2.3	0.7–7.3	0.15
Labor induction for post-term pregnancy	1.9	1.0–3.6	0.044	1.1	0.3–3.7	0.84
Labor induction for premature rupture of membranes	1.7	0.8–3.6	0.14	2.1	0.9–4.7	0.07
Labor induction for diabetes	1.5	0.7–3.2	0.28	1.0	0.4–2.8	0.98
Labor induction for non-diabetic macrosomia	5.6	1.3–25.6	0.03	10.8	2.1–55.9	0.005
Labor induction for fetal reason	2.1	0.6–7.1	0.24	3.2	0.9–11.7	0.75
Labor induction for maternal reason	0.9	0.4–2.0	0.83	1.4	0.4–3.2	0.4
Birthweight ≥4500 g	3.0	1.4–6.7	0.007	3.3	1.3–7.9	0.009

**Table 6:** Risk factors for recurrent cesarean section for failed labor induction or labor dystocia in the subgroup of women with a history of previous failed labor induction^a^ (*n* = 100)

	Unadjusted recurrent failed induction or labor dystocia			Adjusted recurrent failed induction or labor dystocia		
	OR	CI (95%)	*p*-value	OR	CI (95%)	*p*-value
Maternal age ≥ 37	1.1	0.2–6.4	0.92	2.2	0.3–19.3	0.47
IVF	1.1	0.1–12.6	0.74	0.6	0.03–11.1	0.71
Smoking	3.1	0.77–12.7	0.11	10.5	1.5–73.6	0.02
Height < 160 cm	2.7	0.9–8.2	0.08	3.0	0.7–14.5	0.18
BMI ≥ 35	3.1	0.9–10.3	0.07	5.3	1.0–27.4	0.05
More than 2 years from previous CS	1.3	0.5–3.4	0.54	0.9	0.3–3.3	0.92
Diabetes, type 1 or gestational	2.3	0.9–6.1	0.081	1.9	0.4–9.1	0.4
Medication-dependent gestational diabetes	2.3	0.4–12.4	0.32	0.6	0.02–15.3	0.77
Post-term pregnancy (≥42 weeks)	0.9	0.3–2.7	0.88	0.09	0.003–2.3	0.14
Labor induction for post-term pregnancy	1.4	0.5–3.8	0.56	81.9	2.0–3406.7	0.02
Labor induction for premature rupture of membranes	0.9	0.3–2.8	0.84	6.0	0.5–68.9	0.15
Labor induction for diabetes	3.1	0.8–12.7	0.11	19.9	0.5–878.0	0.12
Labor induction for non-diabetic macrosomia	2.2	0.1–37.1	0.58	33.5	0.8–1362.2	0.06
Labor induction for fetal reason	2.3	0.4–12.4	0.32	27.9	1.6–472.8	0.02
Labor induction for maternal reason	1.5	0.4–6.0	0.54	18.0	1.2–268.3	0.04
Birthweight ≥4500 g	2.3	0.3–17.1	0.42	7.0	0.5–90.6	0.14

^a^ of which 92 women with no prior vaginal delivery (92%)

## Discussion

Our results show a relatively high success rate of 73% for TOLAC in women with a history of previous failed labor induction or labor dystocia. Lower vaginal delivery rates of 49–68% following TOLAC for non-progressive labor have previously been reported [11, 12, 15, 19–23]. Our results suggest that the highest rate of repeat CS occurs in women with a history of failed labor induction compared to women with labor dystocia in the first or second stage of labor. Furthermore, every fifth woman with a history of failed labor induction had recurring failed IOL.

The greatest predictor for successful TOLAC is a prior vaginal delivery [4], as also seen in our study. However, two thirds of the women with no prior vaginal delivery in our study also had successful TOLAC. Women undergoing IOL had lower success rate of TOLAC compared to women with spontaneous onset of labor, as reported previously [11, 24]. Increasing maternal age and BMI may propose a greater risk for CS [4, 25–28]. In this study, maternal age ≥ 37 years was associated with repeat CS, and BMI ≥ 35 was associated with recurring failed labor induction or labor dystocia in the subgroup of women with a history of induction failure. In our study, maternal height < 160 cm was associated with an increased risk for repeat CS. This is in line with previous studies suggesting that shorter maternal stature appears an independent risk factor for induction failure, and taller women are more likely to have a vaginal delivery [29, 30]. Also, increasing neonatal birth weight > 4000 g has been shown to increase the risk for a recurrent CS [31–33], which is in agreement with our results.

The overall rate of uterine rupture was low (0.6%) in our study. Higher rates of uterine rupture have been reported following IOL compared to spontaneous onset of labor [24, 34–36], while contradictive results have also been presented [37]. In our study, three of the four uterine ruptures occurred following spontaneous onset of labor. The women with uterine rupture had no prior vaginal delivery, which may increase the risk for uterine rupture [34]. The method of choice for IOL in women with an unfavorable cervix and a history of previous CS is FC [38, 39]. Some women in our study received misoprostol for IOL despite of the uterine scar, even though this is not supported by our clinical guidelines. However, no uterine ruptures occurred in these women.

The rate of postpartum hemorrhage in our study was higher than previously reported [40, 41]. Increased post-partum hemorrhage more often occurred following unsuccessful TOLAC, as also previously reported [24]. The rates of maternal infections were also consistent with previous studies [5, 34, 40]. Higher rate of maternal morbidity and endometritis have been shown to occur in women with an unsuccessful TOLAC compared to women with a successful TOLAC [5], and a similar trend was seen also in our study. Adverse neonatal primary outcomes were not frequent following TOLAC, which is in line with previous studies [23, 40].

The strengths of our study were the relatively large sample size, the systematic and detailed medical records, and standardized labor management protocol in our hospital. The major weaknesses of this study are the retrospective design and not including the women delivering by a planned CS, which may have caused a potential bias. Also, this study may have lacked power to detect possible associations between several important variables. We regret not having the data on estimated fetal weight available in our study, but as a surrogate, we used birthweight. A previous study indicated that a 500 g increase in birthweight in current pregnancy, compared to the pregnancy with labor dystocia, decreases the rate of a successful TOLAC [27]. Furthermore, unfortunately we did not have the data on cervical dilation at the previous CS, as cervical dilation of 7 cm or more has been suggested to increase the likelihood of successful TOL in the subsequent pregnancy [12, 21, 22].

## Conclusions

TOLAC, including both spontaneous and induced labor, is a feasible option for scheduled repeat CS in women with a history of previous failed labor induction or labor dystocia. However, our results suggest that women with no previous vaginal delivery, maternal height < 160 cm, diabetes, and suspected neonatal macrosomia (≥4500 g) may be at increased risk for failed trial of labor. Also, BMI ≥35, smoking, no prior vaginal delivery and induction of labor for reasons other than PROM may be associated with recurring failed induction or labor dystocia.

## Abbreviations

CS: Cesarean section
IOL: Induction of labor
PROM: Premature rupture of membranes
TOLAC: Trial of labor after cesarean
